# Cell line authentication and validation is a key requirement for *Journal of Cell Communication and Signaling* publications

**DOI:** 10.1002/ccs3.70029

**Published:** 2025-06-20

**Authors:** Ralf Weiskirchen, Jamie Almeida, Brahim Chaqour

**Affiliations:** ^1^ Institute of Molecular Pathobiochemistry RWTH University Hospital Aachen Aachen Germany; ^2^ Biosystems and Biomaterials Division National Institute of Standards and Technology Gaithersburg Maryland USA; ^3^ Department of Molecular Ophthalmology Perelman School of Medicine University of Pennsylvania Philadelphia Pennsylvania USA

**Keywords:** cell line authentication, cell line nomenclature, *JCCS*, mycoplasma, STR profiling, validation

## Abstract

Cell lines are essential tools in biomedical research and drug discovery, often substituting for tissues or organs of origin. However, frequent misidentification and cross‐contamination pose major quality control challenges, leading to unreliable data, hindering scientific progress, and impacting clinical translation. Even authenticated cell lines may undergo genetic and phenotypic changes over time, affecting experimental outcomes. To promote transparency, reproducibility, and rigor, the *Journal of Cell Communication and Signaling* (*JCCS*) reaffirms its commitment to best practices in cell line authentication and validation, in alignment with Wiley's publishing ethics. Authors submitting manuscripts must provide comprehensive cell line details, including species, sex, tissue origin, name, and Research Resource Identifier. They are also required to document the source, acquisition date, and authentication methods such as short tandem repeat (STR) profiling and adventitious agent testing, including mycoplasma screening. By enforcing strict guidelines, *JCCS* seeks to improve research integrity, reduce erroneous findings, and enhance reproducibility. This initiative not only strengthens the reliability of published studies but also supports the broader scientific community in accelerating discovery and translating research into clinical advances for better human health.

## Graphical abstract

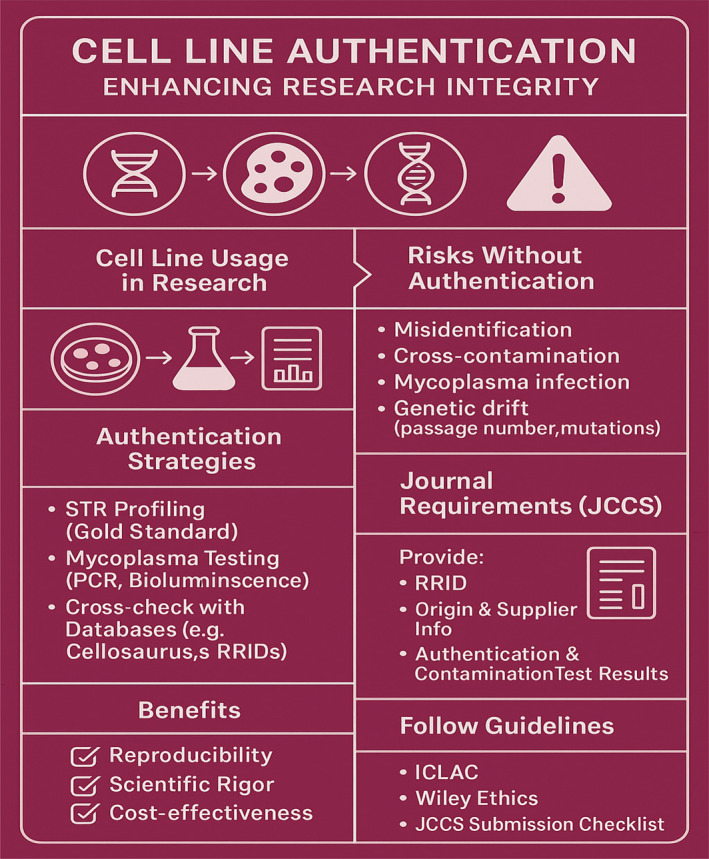

This article emphasizes the importance of proper cell line authentication in biomedical research, highlighting issues like misidentification, contamination, and genetic drift that compromise data reliability and reproducibility. JCCS reinforces its commitment to ethical publishing by requiring detailed documentation and validation of cell lines from authors. These measures aim to enhance research integrity, support scientific progress, and improve clinical translation.

The accurate identification and verification of cell lines used in research studies is essential for ensuring the reliability of published scientific findings and the ability of other investigators to reproduce published data. Cell line identifiers, which consist of short alphanumeric strings, can be cross‐referenced with knowledge databases such as Cellosaurus to verify the identity and origin of any given cell line and identify potential issues such as misidentification, cross‐contamination, or misclassification of cell lines. However, issues like misidentification, microbial contamination (e.g., mycoplasma), cross‐contamination with another cell line, and exceedingly high cell passage number are extensively documented, along with strategies for their prevention. Short tandem repeat (STR) testing is regarded as the gold standard for cell line authentication due to its accuracy, speed, and reliability.^1^ Several reliable methods are also available for mycoplasma detection, such as PCR and bioluminescence.^2^ However, cell lines can undergo many changes as a result of short or prolonged cultivation including increased genetic and phenotypic instability, which may alter cell morphology and gene expression, chromosomal duplication and rearrangements, potential mutations in genomic and mitochondrial DNA, and epigenetic modifications.^3^, ^4^ As a result, there has been a growing emphasis on validating the authenticity and integrity of both experimental and established cell lines prior to publication. Laboratories must establish clear protocols for when and how to conduct these essential tests, evaluate the outcomes, and implement appropriate responses if inconsistencies are found.

*Journal of Cell Communication and Signaling* (*JCCS*) is committed to adhering to best practice guidelines on research integrity and publishing ethics including those for authentication and validation of cell lines used in studies published in the journal. This commitment is in conformity with Wiley publishing ethics guidelines first published in 2006 and revised in 2014. These guidelines aim to improve the rigor and reproducibility of research involving cell lines and ensure the integrity of scientific findings published in the journal. Researchers submitting manuscripts to *JCCS* are encouraged to familiarize themselves with these guidelines to ensure compliance with the updated standards in cell line authentication.

Misidentified or cross‐contaminated cell lines are a widespread and concerning issue that undermines research integrity in the life sciences.^5^, ^6^ When scientists mistakenly use misidentified cell lines, experiments can produce unreliable or irreproducible results, leading to increased costs and potentially misguiding future studies. This not only hinders progress in basic research but also delays the development of clinical applications, negatively affecting patient outcomes and the scientific community as a whole.^7^, ^8^, ^9^ The *JCCS* cell authentication guidelines highlight the importance of transparency, reproducibility, and a more thorough evaluation of cell lines, demonstrating the research community's dedication to enhancing standards and uphold scientific rigor.

Despite rapidly advancing biotechnologies, including sophisticated genomic and proteomic tools, the problem of cell line misidentification persists. Over the years, numerous studies have documented the prevalence of contaminated and misidentified lines, each of which has the potential to invalidate otherwise carefully designed studies.^6^ A key concern is that time and resources are wasted on experiments that are based on shaky foundations. The consequences of using misidentified cell lines can be far‐reaching, including unwarranted conclusions, reduced confidence in the scientific literature, and, in the case of potentially translational or clinical investigations, inefficient use of resources that could otherwise be directed toward viable therapeutic pathways.

The updated *JCCS* submission guidelines now require authors to provide critical information for each cell line used in a study. This information includes

1. The species, sex determination, tissue of origin, official cell line name, and a Research Resource Identifier (RRID) for immortalized cell lines. The RRID system has been developed to track reagents such as cell lines and antibodies consistently throughout the scientific literature. By assigning unique identifiers, databases and publishers can trace the origin of a cell line and cross‐reference its authentication data, helping to prevent the inadvertent use of misidentified lines, improve reproducibility, and lower the incidences of problematic cell lines in papers.^10^, ^11^
2. Authors must also report the source or supplier of the cell line and the date it was obtained. Cell lines can genetically diverge over time, so knowing when a laboratory acquired a particular cell line is essential for accurate record‐keeping and encouraging regular authentication testing.
3. Equally important, authors must describe their cell line authentication procedures, such as STR profiling or other molecular methods used to verify cell line identity, including DNA barcoding for nonhuman cell lines. *JCCS* also requires investigators to specify tests used to rule out contamination, particularly mycoplasma, a common contaminant in cell culture. These authentication details and test results must be documented at the time of manuscript submission. If any information is unknown, authors must state this and provide a rationale for the use of the cell line. All papers submitted to *JCCS* must meet these standards in accordance with the journal's commitment to rigorous, high‐quality methods.

The *JCCS* submission guidelines listed above are similar to those described in the NIH guidelines for applications and funding (reference https://grants.nih.gov/grants/guide/notice‐files/NOT‐OD‐15‐103.html) and the guidelines for reporting (reference https://grants.nih.gov/policy‐and‐compliance/policy‐topics/reproducibility) that many journals have already adopted. Qualifying reagents used in research, including identity testing of cell lines and the presence of adventitious agents, are paramount in maintaining transparency and improving reproducibility in the scientific community.

For guidance on best practices for authentication, authors can consult the resources of the International Cell Line Authentication Committee (ICLAC).^12^ The members of this global organization advise scientists on cell line identification, contamination control, and database development to address the challenge of misidentification. ICLAC provides guidelines and recommendations that outline authentication processes from sample collection to data interpretation. Following these protocols can improve the reliability of experiments, reduce the risk of cell contamination, and overcome the challenges posed by misidentified cell lines. For a comprehensive overview of currently recognized misidentified cell lines, please refer to Table 1, which is continuously updated by the ICLAC to reflect the latest knowledge. A modified list of the listed cell lines sorted by the claimed species can be found elsewhere.^14^

**TABLE 1:** Register of misidentified cell lines (in alphabetical order).^a^

	Most frequently used name of cell line^b^
Nos.	1.1B4; 1.1E7; 1‐1ras1000; 1‐1src; 1E8; 2008/C13*5.25; 222; 2474/90; 2563; 28SC‐ES; 2957/90; 3051/80; 3AB‐OS; 41M; 5–8F; 6–10B
A	A172TR3; ACC2; ACC3; ACCM; ACCNS; ACCS; ACN; ADLC‐5M2; *Aedes aegypti*, Suitor's clone; *Aedes vexans* culture; AG‐F; AKI; ALVA‐101; ALVA‐31; ALVA‐41; ALVA‐55; AO; Ao38; ARO81‐1; AV3; AZ521
B	BALB/3T3 A31‐1‐1; BALB/3T3 A31‐1‐13; BCaP‐37; BCC1/KMC; BE‐13; BEL‐7402; BEL‐7404; BGC‐823; Bhas42; BHP 10‐3; BHP 14‐9; BHP 15‐3; BHP 17‐10; BHP 18–21; BHP 2–7; BHP 5–16; BHP 7–13; BIC‐1; BLIN‐1; BM‐1604; BrCA 5; BSCC‐93; BT‐B
C	C16; C‐433; CAC2; CaES‐17; CaMa; CaOV; Caov‐2; CaVe; CCL3; CCO; CCO‐SFM; CGTH‐W‐1; CH1; CH1‐cisR; Chang liver; CHB; CHP‐234; Clom 15; Clone 1‐5c‐4; Clone 1A; CM‐319; CMP; CMPII C2; CNDT2; CNE‐1; CNE‐2; CO; COLO‐38; COLO‐587; COLO‐677; COLO‐775; COLO‐818; CoLo‐TC; *Culiseta inornata*
D	D‐11; D18T; D‐54 MG; D98/AH; D98/AH2 Clone B; DAMI; DAPT; DD; Det30A; Detroit 98/AG; Detroit 98/AH‐2; Detroit 98/AH‐R; Detroit 98s; Detroit‐6; Detroit‐98; DM12; DM14; DRO90‐1; DuPro‐1
E	E006AA; E006AA‐hT; EB33; EC9706; eCAS; ECC‐1; ECTC; ECV‐304; ED27; EEK; EH; EJ‐1; Ej138; EL 1; ElCo; EPC; EPLC3‐2M1; EPLC‐65; ESP1; ETK‐1; EU‐1; EU‐7; EUE; EVLC2
F	F2‐4E5; F255A4; F2‐5B6; FB2; Fitz‐HSA; FL; Flow 13,000; Flow 5000; Flow 6000; Flow 7000; FQ; FRI‐SpIm‐1229
G	G‐11; GHE; Girardi heart; GLC‐82; GM1312; GOS‐3; GPS‐M; GPS‐PD; GREF‐X; GR‐M; GT3TKB
H	H‐494; H7D7A; H7D7B; H7D7BD5; H7D7C; H7D7D; HAC15; HAC‐84; HAG; HAPI; HBC; HBL‐100; HBT‐3; HBT‐39b; HBT‐E; HCC60; HCE; HCu‐10; HCu‐18; HCu‐22; HCu‐27; HCu‐33; HCu‐37; HCu‐39; HCV‐29Tmv; HEC‐155; HEC‐180; HEK; HEK/HRV; HEL‐R66; Hep2; HEp‐2; Hep‐2C; Hepa‐T1; HES; HHUA; HIMEG‐1; HKB‐1; HKMUS; HKMUS‐SF; HKTN‐2; HL111783; HMV‐1; HNOS; HO‐8910; HO‐8910PM; HONE‐1; HPB‐MLT; HPC‐36M; hPTC; HROBML03; Hs 677.St; HSC‐41; HSG; HSG‐AZA1; HSG‐AZA3; HSGc‐C5; HS‐SULTAN; HSY; hTERT‐EEC; Hu1734; Hu456; Hu549; Hu609; Hu609Tmv; Hu961a, Hu961t; Huker; HuKo39; HuL‐1; Hut
I	IMC‐2; IMC‐3; IMC‐4; ImKC; Intestine 407; IPDDC‐A2; IPRB; IPRI‐OL‐11; IPRI‐OL‐7; IPTP/98; IST‐1; IZD‐MB‐0503
J	J‐111; J96; JCA‐1; JHC; JHPI‐1 clone 16; JHT; JHU012; JHU013; JHU019; JHU028; JMAR; JOSK‐I; JOSK‐K; JOSK‐M; JOSK‐S; JROECL 47; JROECL 50; JTC‐17; JTC‐3
K	K051; K1; K2; K5; KAK1; KAT10; KAT4; KAT5; KAT50; KAT7; KB; KB‐3‐1; KB‐V1; KCI‐MOH1; KKU‐213B; KKU‐213C; KM20; KM20L2; KM3; KM‐3; KMS‐21‐BM; KMT‐2; KNS‐89; KOSC‐3; KP‐1N; KPB‐M15; KPL‐1; KP‐P1; KSY‐1; KU7; KU‐YS
L	L‐02; L‐132; L‐41; LAH1; LAH2; LC5; LC5‐HIV; LED‐Ti; LF‐BK; LFBK‐alphaVbeta6; LLC‐15MB; LN‐319; LN‐443; LN‐464; LR10.6; LT‐1; LTEP‐a2; LU; LU 106; Lu‐130
M	M10T; M4A4; M4A4 GFP; M4A4 LM3‐2 GFP; M4A4 LM3‐4 CL16 GFP; MA‐1; MA‐104; MA‐111; MA‐160; MARC‐145; Mash‐1; MaTu; MC‐4000; McCoy; MCF‐7/AdrR; MDA‐MB‐435; MDA‐MB‐435S; MDA‐N; MDS; MEL‐HO; MEL‐WEI; MGC‐803; MGH‐U1; MGH‐U2; MHH‐225; Minnesota EE; MKB‐1; MKN28; MOBS‐1; MOC2‐10; MOLT‐15; MPanc‐96; MRO87‐1; MS; MT‐1; MT‐3; MUM2C; MUTZ‐1; MV522
N	NC‐37; NCC16; NCI‐H1264; NCI‐H1304; NCI‐H1514; NCI‐H157; NCI‐H1622; NCI‐H1870; NCI‐H249; NCI‐H513; NCI‐H592; NCI‐H60; NCI‐H630; NCI‐H738; NCOL‐1; NCTC 2544; NCTC 3075; ND‐1; NM2C5; NM2C5 GFP; NOI‐90; NOK‐SI; NOSE06; NOSE07; NPA'87; NS‐3
O	OCM‐1; OCM‐3; OCM‐8; OCUM‐6; OE; OF; OLGA‐PH‐J/92; ONCO‐DG‐1; OS 187; OST; OU‐AML‐1; OU‐AML‐2; OU‐AML‐3; OU‐AML‐4; OU‐AML‐5; OU‐AML‐6; OU‐AML‐7; OU‐AML‐8; OV2008; Ovary1847; OVMIU
P	P1‐1A3; P1‐4D6; P39/TSUGANE; Panc 01.28; Panc 06.03; PBEI; PC‐93; PCI‐22A; PCI‐22B; PCI‐3; PC‐MDS; PEAZ‐1; PH; PH61‐N; PLB‐985; PMF‐ko14; PPC‐1; PSV811
Q	QGY‐7701; QGY‐7703; QSG‐7701
R	R1; RAMAK‐1; RB; RBHF‐1; RC‐2A; RED‐3; REH‐6; REPC; RERF‐LC‐MA; RERF‐LC‐OK; RGC‐5; RM‐10; RML‐15; RMUG‐L; RO‐D81‐1; RO‐H85‐1; RPMI‐4788; RPMI‐6666; RPTC‐1; RS‐1; RTSG; RY
S	SA4; SAM‐1; SAML‐1; SBC‐2; SBC‐7; SC; SCCTF; SCLC‐16H; SCLC‐24H; SEG‐1; SF767; SGC‐7901; SH‐2; SH‐3; SJPL; SK‐GT‐5; SK‐MG‐1; SK‐N‐MC; SK‐OV‐4; SK‐OV‐6; SKW 6.4; SKW‐3; SLK; SLR20; SLR24; SMMC‐7721; SNB‐19; SNG‐M; SNU‐1958; SPC‐A1; SPC‐BM‐36; SPI‐801; SPI‐802; SpR; SQ‐5; SR‐91; SU‐DHL‐9; SUNE1; SUNE2; SW‐527; SW‐598; SW‐608; SW‐613; SW‐732; SW‐733
T	T1; T‐1; T‐33; T404; T406; T409; T‐9; Tca8113; TCO‐1; TDL‐1; TDL‐2; TDL‐3; TDL‐4; TE‐12; TE‐13; TE‐2; TE‐3; TE671; TE671 Subline No.2; TE‐7; TEC61; TI‐1; TK‐1; TMH‐1; TMM; TSCCa; TSU‐Pr1; Tu‐138; Tu‐158LN; Tu‐159; Tu‐167; Tu‐182; Tu‐212; Tu‐212LN; TuWi
U	U‐118 MG; UCDK9B1; UCDK9B2; UCDK9B3; UCDK9B4; UCDK9B5; UM‐UC‐2; UM‐UC‐3‐GFP; UPES/C; UPHHJA; UTMB‐460
V	VC312R
W	WiDr; WISH; Wong–Kilbourne derivative; WRL 68; WSU‐ALCL; WSU‐CLL
Y	YAA; YAP; YJ; YMB‐1; YMB‐1‐E
Z	Z‐HL16C

^a^ The names of misidentified cell lines were taken from the ICLAC register of misidentified cell lines, version 13, released on April 26, 2024, available at Ref.^12^

^b^ It is important to note that some of the cell lines listed may have synonyms. Additional information on cell lines can be found in the Cellosaurus knowledge resource.^13^

The Cellosaurus database is an essential resource for tracking cell lines.^15^ Maintained by the Swiss Institute of Bioinformatics, Cellosaurus is a comprehensive knowledge base, encompassing thousands of cell lines from various species. The latest version of this database (release April 52, 2025) contains information on 163,868 cell lines (e.g., 121,295 from human, 29,536 from mouse, 3115 from rat) commonly used in biomedical research. Each entry typically includes information on nomenclature, known synonyms, and cross‐references to other databases such as the RRID portal.^16^ Scientists and journal editors can consult Cellosaurus to validate the known properties of a particular cell line, check whether it has been flagged for contamination or misidentification, and resolve synonyms or conflicting names. Similarly, the NCBI database of misidentified cell lines serves as a valuable resource, listing cell lines known to be misidentified or contaminated.^17^

By establishing strict requirements for cell line identification, contamination testing, and documentation, *JCCS* sends a clear signal that high quality and reproducible science is not only encouraged but required. Promoting a culture of accountability in cell culture experiments will, over time, reduce the prevalence of erroneous data, protect the integrity of scientific publications, and strengthen the overall impact of research on cell communication and signaling. Although these steps are an important milestone, continued support for initiatives such as ICLAC, the RRID system, the NCBI misidentified cell line database, and the Cellosaurus database will ensure that scientists worldwide have the tools and guidance they need to maintain rigorous standards. Together, these efforts pave the way for research that is both credible and impactful, ultimately advancing our collective understanding of biological processes and improving animal and human health.

The adoption of these new guidelines by *JCCS* underscores the importance of proactive measures at multiple levels of scientific endeavor. Beyond academic institutions and funding agencies, journals have a vital role to play in reinforcing good laboratory practice. In the wider context, peer reviewers and editors must rigorously scrutinize submissions to ensure compliance with these standards and present a united front that prioritizes rigor and transparency. Ultimately, the responsibility for enforcing these safeguards against misidentified or contaminated cell lines does not rest solely with *JCCS* or any other single journal or organization. The entire scientific community must embrace these principles to maintain public trust, optimize resource use, and accelerate meaningful discoveries. Whether a researcher works in a small academic lab or a large pharmaceutical company, properly authenticated cell lines ensure that hypotheses are tested and data are generated under conditions of maximum reliability. The result is a more trustworthy literature, one in which results can be reliably reproduced, extended, and translated into clinical benefit.

## AUTHOR CONTRIBUTIONS

All authors were equally involved in the preparation of this article.

## CONFLICT OF INTEREST STATEMENT

Brahim Chaqour is the editor‐in‐chief of *JCCS.* Jamie Almeida is Chair of International Cell Line Authentication Committee (ICLAC). Ralf Weiskirchen is member of ICLAC.

## ETHICS STATEMENT

No ethics approval needed.

## Data Availability

The data that support the findings of this study are available from the corresponding author upon reasonable request.
